# *GNA11* Variants Identified in Patients with Hypercalcemia or Hypocalcemia

**DOI:** 10.1002/jbmr.4803

**Published:** 2023-04-18

**Authors:** Sarah A. Howles, Caroline M. Gorvin, Treena Cranston, Angela Rogers, Anna K. Gluck, Hannah Boon, Kate Gibson, Mushtaqur Rahman, Allen Root, M. Andrew Nesbit, Fadil M. Hannan, Rajesh V. Thakker

**Affiliations:** ^1^ Academic Endocrine Unit, Radcliffe Department of Medicine University of Oxford Oxford UK; ^2^ Nuffield Department of Surgical Sciences University of Oxford Oxford UK; ^3^ Oxford Molecular Genetics Laboratory Churchill Hospital Oxford UK; ^4^ Department of Endocrinology Northwick Park Hospital, North West London Hospitals NHS Trust Harrow UK; ^5^ Department of Endocrinology John Hopkins All Children's Hospital St. Petersburg Florida USA; ^6^ Biomedical Sciences Research Institute University of Ulster Coleraine UK; ^7^ Nuffield Department of Women's & Reproductive Health University of Oxford Oxford UK; ^8^ National Institute for Health Research Oxford Biomedical Research Centre Oxford UK; ^9^ Present address: Institute of Metabolism and Systems Research, University of Birmingham, and Centre for Endocrinology, Diabetes and Metabolism, Birmingham Health Partners Birmingham UK

**Keywords:** CELL/TISSUE SIGNALING‐ENDOCRINE PATHWAYS, CALCIUM‐SENSING RECEPTOR, G PROTEIN‐COUPLED RECEPTORS, FAMILIAL HYPOCALCIURIC HYPERCALCEMIA

## Abstract

Familial hypocalciuric hypercalcemia type 2 (FHH2) and autosomal dominant hypocalcemia type 2 (ADH2) are due to loss‐ and gain‐of‐function mutations, respectively, of the *GNA11* gene that encodes the G protein subunit Gα11, a signaling partner of the calcium‐sensing receptor (CaSR). To date, four probands with FHH2‐associated Gα11 mutations and eight probands with ADH2‐associated Gα11 mutations have been reported. In a 10‐year period, we identified 37 different germline *GNA11* variants in >1200 probands referred for investigation of genetic causes for hypercalcemia or hypocalcemia, comprising 14 synonymous, 12 noncoding, and 11 nonsynonymous variants. The synonymous and noncoding variants were predicted to be benign or likely benign by in silico analysis, with 5 and 3, respectively, occurring in both hypercalcemic and hypocalcemic individuals. Nine of the nonsynonymous variants (Thr54Met, Arg60His, Arg60Leu, Gly66Ser, Arg149His, Arg181Gln, Phe220Ser, Val340Met, Phe341Leu) identified in 13 probands have been reported to be FHH2‐ or ADH2‐causing. Of the remaining nonsynonymous variants, Ala65Thr was predicted to be benign, and Met87Val, identified in a hypercalcemic individual, was predicted to be of uncertain significance. Three‐dimensional homology modeling of the Val87 variant suggested it was likely benign, and expression of Val87 variant and wild‐type Met87 Gα11 in CaSR‐expressing HEK293 cells revealed no differences in intracellular calcium responses to alterations in extracellular calcium concentrations, consistent with Val87 being a benign polymorphism. Two noncoding region variants, a 40bp‐5'UTR deletion and a 15bp‐intronic deletion, identified only in hypercalcemic individuals, were associated with decreased luciferase expression in vitro but no alterations in *GNA11* mRNA or Gα11 protein levels in cells from the patient and no abnormality in splicing of the *GNA11* mRNA, respectively, confirming them to be benign polymorphisms. Thus, this study identified likely disease‐causing *GNA11* variants in <1% of probands with hypercalcemia or hypocalcemia and highlights the occurrence of *GNA11* rare variants that are benign polymorphisms. © 2023 The Authors. *Journal of Bone and Mineral Research* published by Wiley Periodicals LLC on behalf of American Society for Bone and Mineral Research (ASBMR).

## Introduction

Familial hypocalciuric hypercalcemia (FHH) and autosomal dominant hypocalcemia (ADH) are genetically heterogeneous autosomal dominant disorders of calcium homeostasis. FHH results in a lifelong elevation in serum calcium concentrations in association with a reduced urinary calcium excretion, while serum calcium concentrations are low in ADH and associated with hypercalciuria in 10% of patients.^(^ ^1^, ^2^ ^)^ In most patients, parathyroid hormone (PTH) levels remain within the normal range, indicating that these disorders are due to impaired calcium sensing, which results in an alteration in the “set point” for the regulation of PTH release.^(^ ^3^ ^)^ In 65% of cases, FHH is caused by loss‐of‐function mutations of the calcium‐sensing receptor (CaSR) (FHH type 1, FHH1), while over 90% of cases of ADH are caused by gain‐of‐function mutations in the CaSR (ADH type 1, ADH1).^(^ ^4^, ^5^, ^6^, ^7^ ^)^ The CaSR is a family C G‐protein‐coupled receptor (GPCR) that plays a pivotal role in the parathyroid and renal regulation of extracellular calcium concentrations ([Ca^2+^_o_]). Loss‐ and gain‐of‐function mutations in the G‐protein alpha subunit 11 (Gα_11_), which is a major downstream signaling partner of the CaSR, cause FHH2 and ADH2, respectively. FHH3, which represents <10% of the FHH cases, is due to missense mutations in the adaptor protein 2 sigma subunit.^(^ ^8^ ^)^ The remaining cases of FHH and ADH are due to an unknown genetic abnormality.^(^ ^8^, ^9^, ^10^, ^11^, ^12^ ^)^

**Fig. 1:**
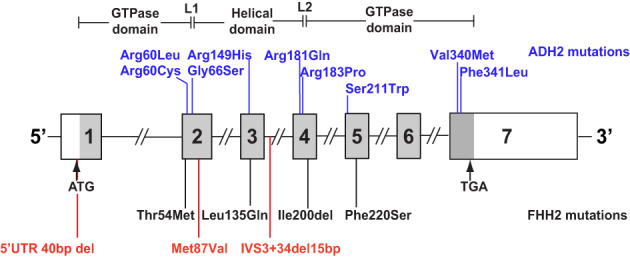
Schematic representation of genomic organization of human *GNA11* gene showing locations of FHH2‐ and ADH2‐causing mutations. The GNA11 gene consists of seven exons with the start (ATG) and stop (TGA) codons located in exons 1 and 7, respectively. The GTPase domain (encoded by exon 1, 5′ portion of exon 2, 3′ portion of exon 4 and exons 5 to 7) is connected to the helical domain (encoded by the 3′ portion of exon 2, exon 3, and 5′ portion of exon 4) by the linker 1 (L1) and linker 2 (L2) peptides. The previously reported ADH2‐associated gain‐of‐function and FHH2‐associated loss‐of‐function mutations are shown in blue and black, respectively. The identified *GNA11* variants studied in this manuscript are shown in red. Coding regions are shaded gray, and untranslated regions are represented by open boxes.

Gα_11_, a member of the G_q/11_ class of G‐proteins, enhances phospholipase C (PLC) activity, which leads to the formation of inositol 1,4,5‐trisphosphate (IP_3_), which induces increases in intracellular Ca^2+^ concentrations ([Ca^2+^_i_]).^(^ ^13^, ^14^ ^)^ These signal transduction events allow the CaSR to respond to small fluctuations in the prevailing [Ca^2+^]_o_ and to induce alterations in PTH secretion and urinary calcium excretion.^(^ ^6^ ^)^ To date, only four FHH2 mutations (Thr54Met, Leu135Gln, Ile200del, Phe220Ser) and nine ADH2 mutations (Arg60Leu, Arg60Cys, Gly66Ser, Arg149His, Arg181Gln, Arg183Pro, Ser211Trp, Val340Met, Phe341Leu) have been described (Fig. 1*A*).^(^ ^15^, ^16^ ^)^ Following the identification of FHH2 and ADH2 in 2013, we have undertaken *GNA11* mutational analysis in additional hypercalcemic and hypocalcemic probands.^(^ ^6^ ^)^ Here, we describe the identification and classification of *GNA11* variants over this 10‐year period and the functional characterization of three rare *GNA11* variants of likely benign or uncertain significance (VUS).^(^ ^17^ ^)^

## Materials and Methods

### Patient population

Patients referred to the Oxford Molecular Genetics Laboratory and the Academic Endocrine Unit, University of Oxford, for investigation of a genetic cause for their hypercalcemia or hypocalcemia between 2013 and 2022 were included, as well as data and samples from participants in related ethically approved research.

### Study approval

This analysis of data from clinical care was approved by the Joint Research Office University of Oxford and the Oxford University Hospitals NHS Foundation Trust and judged to be a description of the findings of clinical service. As such, it does not require research governance. Research findings are from the conduct of a study with ethical approval from the London (UK)‐Central Research Ethics Committee (MREC/02/2/93).

### DNA sequence analysis

Gene analyses were performed using leucocyte DNA as follows. Hypercalcemic patients had stepwise analysis of genes known to be implicated in the etiology of primary hyperparathyroidism and hypercalcemic disorders such as *MEN1*, *CDC73*, *CDKN1B*, *CASR*, *AP2S1*, *GCM2*, *GNA11*, and *RET* or were referred for analysis of a gene panel comprising these eight genes.^(^ ^7^, ^18^ ^)^ Hypocalcemic patients had stepwise analysis of hypoparathyroid genes such as *AIRE*, *CASR*, *GNA11*, *GCM2*, and *GATA3* or were referred for analysis of a gene panel comprising *AIRE*, *CASR*, *GATA3*, *GCM2*, *GNA11*, *PTH*, and *TBCE*.^(^ ^7^ ^)^ If required, analysis of *GNA11* and *AP2S1* was also undertaken in which exons 1–7, the 12 exon‐intron boundaries and 5′ untranslated region (UTR) of *GNA11* (NM_002067) (Fig. 1), and exons 1–5, the 8 exon‐intron boundaries and 5′ UTR of *AP2S1* (NM_004069), were sequenced using gene‐specific primers (Sigma‐Aldrich, St. Louis, MO, USA), as previously reported.^(^ ^4^, ^6^, ^9^ ^)^ The genome aggregation database (https://gnomad.broadinstitute.org/) was examined for the presence of sequence variants.

### Classification of likely pathogenicity of *GNA11* variants

The likely pathogenicity of *GNA11* variants was assessed in accordance with the Association for Clinical Genomic Science (ACGS) best practice guidelines (https://www.acgs.uk.com/quality/best-practice-guidelines/).^(^ ^17^ ^)^ Variants were characterized in silico using Alamut Visual version 2.11 (Interactive Biosoftware, Rouen, France).

### Protein sequence alignment and three‐dimensional modeling of Gα11 structure

Protein sequences of Gα11 orthologs and paralogs were aligned using ClustalOmega.^(^ ^19^ ^)^ SIFT, MutationTaster, and MetaLR were used to predict the effect of amino acid substitutions.^(^ ^20^, ^21^, ^22^ ^)^ Secondary structures of Gα11 were compared using Spider2.^(^ ^23^ ^)^ Gα11 three‐dimensional modeling was undertaken using the reported three‐dimensional structure of Gαq in complex with guanosine diphosphate (GDP) (Protein Data Bank accession no. 3AH8)^(^ ^24^ ^)^ as full‐length structures of Gα11 are not available and Gα11 shares 90% identity at the amino acid level with Gαq. Molecular modeling was performed using the PyMOL Molecular Graphics System (version 1.2r3pre, Schrodinger, LL Pymol).^(^ ^6^ ^)^

### Cell culture and transfection

Wild‐type (WT) and mutant *GNA11* (pBI‐CMV2‐*GNA11*) expression constructs used in Ca^2+^_i_ assays were generated as previously described^(^ ^6^, ^8^ ^)^ and transiently transfected into HEK293 cells stably expressing the CaSR (HEK293‐CaSR),^(^ ^8^ ^)^ along with empty vector pBI‐CMV2, using Lipofectamine 2000 (Life Technologies Carlsbad, CA, USA).^(^ ^4^, ^6^, ^8^, ^9^ ^)^ The bidirectional vector pBI‐CMV2 (Clontech, Mountain View, CA, USA) was used since it expresses GFP and Gα11 at equivalent levels, thereby enabling GFP expression to be used as a surrogate for Gα11 expression.^(^ ^6^, ^8^ ^)^ Mutations were introduced by site‐directed mutagenesis using the Quikchange Lightning Site‐directed Mutagenesis kit (Agilent Technologies, Santa Clara, CA, USA). For luciferase assays, a 1542‐bp region of the *GNA11* promoter and 5'UTR (residues −1542 to −1 with respect to the translation‐initiation codon [ATG]) were inserted upstream of the luciferase reporter gene (*luc2*) in the pGL4.10 (Promega, Fitchburg, WI, USA) expression vector (Geneart, LifeTechnologie, Bayern, Germany). HEK293‐CaSR cells were maintained in DMEM‐Glutamax medium (Thermo Fisher Scientific, Waltham, MA, USA) with 10% fetal bovine serum (Gibco, Thermo Fisher Scientific) and 400 μg/mL geneticin (Thermo Fisher Scientific) at 37°C, 5% CO_2_. Epstein–Barr virus (EBV)‐transformed lymphoblastoid cell lines were established from peripheral blood cells using methods described previously^(^ ^25^ ^)^ and maintained in RPMI‐1640 media (Thermo Fisher Scientific) supplemented with 20% fetal bovine serum (Gibco, Thermo Fisher Scientific) and 100 U/mL penicillin and 100 μg/mL streptomycin (Gibco, Thermo Fisher Scientific). Cells were imaged using an Eclipse E400 fluorescence microscope and images captured using a DXM1200C digital camera and NIS Elements software (Nikon, Tokyo, Japan).^(^ ^4^, ^6^, ^8^ ^)^

### Intracellular calcium measurements

The effects of transfected WT and mutant Gα11 were functionally assessed using a flow cytometry‐based assay that measures alterations in [Ca^2+^]_i_ in response to changes in [Ca^2+^]_o_, as previously described.^(^ ^4^, ^6^, ^8^, ^9^ ^)^ Briefly, 48 hours after transfection, the cells were harvested, washed in calcium‐ and magnesium‐free Hanks' balanced salt solution (HBSS) (Invitrogen, Waltham, MA, USA), and loaded with 1 μg/mL indo‐1‐acetoxymethylester (Indo‐1‐AM) (Molecular Probes, Invitrogen) for 1 hour at 37°C. After removal of free dye, the cells were resuspended in calcium‐ and magnesium‐free HBSS and maintained at 37°C. Transfected cells, in suspension, were stimulated by sequentially adding Ca^2+^ to the Ca^2+^‐ and Mg^+^‐free HBSS to increase [Ca^2+^]_o_ in a stepwise manner from 0 to 15 mM and then analyzed on a MoFlo modular flow cytometer (Beckman Coulter, Indianapolis, IN, USA) by simultaneous measurements of GFP expression (at 525 nm), Ca^2+^_i_ bound Indo‐1AM (at 410 nm), and free Indo‐1AM (ie, not bound to Ca^2+^_i_) (at 485 nm), using a JDSU Xcyte UV laser (Coherent Radiation, Santa Clara, CA, USA), on each cell at each [Ca^2+^]_o_, as described.^(^ ^4^, ^6^, ^8^, ^9^ ^)^ The baseline Indo‐1AM fluorescence ratio was measured for 2 minutes, the fluorescence ratio versus time recorded, and data collected for 2 minutes at each [Ca^2+^]_o_. Cytomation Summit software was used to determine the peak mean fluorescence ratio of the transient response after each individual stimulus expressed as a normalized response, as previously reported.^(^ ^4^, ^6^, ^8^, ^9^ ^)^ Nonlinear regression of concentration‐response curves was performed with GraphPad Prism (GraphPad, San Diego, CA) using the normalized response at each of nine different [Ca^2+^]_o_ for each separate experiment for the determination of the EC_50_ (ie, [Ca^2+^]_o_ required for 50% of the maximal response). The mean EC_50_ from four separate transfection experiments was used for statistical comparison using the *F*‐test.

### Western blot analysis

Following flow cytometry analysis, cells were pelleted and used for Western blot analyses. Flow cytometry cells and lymphoblastoid cells were lysed in NP40 lysis buffer (50 mM Tris HCl pH 7.4, 1 mM EDTA, 150 mM NaCl, protease inhibitors), resuspended in Laemmli buffer, boiled, and separated on 12% sodium dodecyl sulfate‐polyacrylamide gel electrophoresis (SDS‐PAGE) gels. Following transfer to polyvinylidene difluoride membrane (Amersham), blots were blocked in 5% nonfat milk/TBS‐T, then probed with primary antibodies. The following antibodies were used for Western blot analysis: anti‐calnexin (1:1000, Millipore, AB2301), anti‐Gα_11_ (1:1000, SantaCruz, sc‐390382), and anti‐GFP (1:1000, SantaCruz, sc‐9996). Blots were visualized using Immuno‐Star WesternC kit (BioRad) on a BioRad Chemidoc XRS+ system. Blots were stripped with Restore Western blot stripping buffer (Thermo Fisher Scientific) and blocked in 5% marvel/TBS‐T between probing with each primary antibody. Densitometric analysis was performed using ImageJ analysis software (version 1.46; rsb.info.nih.gov/ij/), and statistical analyses were performed using one‐way ANOVA with Dunnett's multiple comparisons test.^(^ ^6^ ^)^ Expression levels of Gα11 were normalized to calnexin expression and expressed relative to the mean relative density of the three unaffected cell lines.

### Quantitative PCR (qPCR)

RNA was extracted from EBV‐transformed lymphoblastoid cells using the MirVana (Ambion, Austin, TX, USA) kit and 1 μg used to generate cDNA using Quantiscript reverse transcriptase in the Quantitect Reverse Transcription kit (Qiagen, Venlo, The Netherlands). qPCR was performed using the Rotorgene SYBR Green Kit in quadruplet in four biological replicates for each EBV‐transformed lymphoblastoid cell line on a Rotorgene 5 (Qiagen).^(^ ^26^ ^)^ Gene‐specific primers were obtained from Quantitect (Qiagen). Each qPCR sample was normalized to levels of the geometric mean of four housekeeper genes, *CANX*, *PGK1*, *TBP1*, and *GAPDH*. Threshold cycle (*C*_T_) values were obtained from the start of the log phase on Rotorgene Q software and analyzed using the Pfaffl method.^(^ ^27^ ^)^ Data for each cell line were normalized to the average of the three cell lines from unaffected control individuals, and statistical analyses were performed by Kruskal–Wallis ANOVA with Dunn's multiple comparisons test.

### Luciferase assays

Luciferase reporter constructs were transiently transfected into HEK293‐CaSR cells in 24‐well plates along with pRL control vector. Cells were lysed at 18 hours after transfection and assayed for luciferase activity using the Dual Luciferase Reporter Assay (Promega), as previously described.^(^ ^5^, ^28^ ^)^ The Turner Biosystems Veritas Microplate Luminometer was used to detect luminescence.^(^ ^5^, ^28^ ^)^ Results are presented as mean ± SEM. Statistical analyses were performed using a one‐way ANOVA with Dunnett's multiple comparisons test, with a single pooled variance.

## Results

### Identification of *GNA11* variants in hypercalcemic and hypocalcemic probands

Between 2013 and 2022, DNA sequence analysis identified 37 different *GNA11* variants in 1226 probands referred for investigation of a genetic cause of their hypercalcemia (*n* = 1014) or hypocalcemia (*n* = 212) (Table 1). Circulating PTH concentrations were available for 151 hypercalcemic probands. The PTH values were either inappropriately normal or raised in >95% of hypercalcemic probands, consistent with a diagnosis of FHH or primary hyperparathyroidism. Circulating PTH concentrations were also available for 31 hypocalcemic probands. PTH values were either inappropriately normal or low in all hypocalcemic probands, consistent with ADH or hypoparathyroidism. *GNA11* variants comprised 14 synonymous, 12 noncoding, and 11 nonsynonymous variants. All synonymous and noncoding variants were predicted to be benign or likely benign by in silico analysis, with five (c.189C>T, c.771C>T, c.[771C>T]+[771C>T], c.771T>C, c.[771T>C]+[771T>C]), and three (c.321 + 9G>A, c.736‐6C>T, c.889 + 8G>C), respectively, also occurring in both hypercalcemic and hypocalcemic individuals; given that pathogenic variants would be expected to cause either hyper‐ or hypocalcemia, but not both, this is consistent with their benign nature in relation to calcium homeostasis (Table 1). Of the remaining noncoding variants, c.‐43_‐4del and c.321 + 35_321 + 49del (Table 1, Fig. 1) occurred only in hypercalcemic probands, and the c.321 + 35_321 + 49del variant occurred in a homozygous state in two hypercalcemic probands (07/14 and 17/09, Table 2), one of which also harbored the c.‐43_‐4del variant in a heterozygous state (07/14, Table 2). Nine of the nonsynonymous variants (Thr54Met, Arg60His, Arg60Leu, Gly66Ser, Arg149His, Arg181Gln, Phe220Ser, Val340Met, Phe341Leu), identified in 13 individuals, had been previously reported in FHH2 or ADH2 probands.^(^ ^15^ ^)^ One of the remaining nonsynonymous variants (Ala65Thr) was predicted by in silico analysis to be benign, and the other variant, Met87Val, identified in a hypercalcemic proband (19/12b, Table 2) was predicted to be a VUS (Table 1). The missense Met87Val VUS and the c.‐43_‐4del and c.321 + 35_321 + 49del variants were therefore selected for further investigation to determine whether they were pathogenic and likely contributing to the phenotype of the probands.

**Table 1:** *GNA11* Variants Identified in >1200 Hypercalcemic and Hypocalcemic Probands

Nucleotide change	Predicted consequence	No. hypercalcemic probands	No. hypocalcemic probands
**Benign/likely benign *GNA11* variants**			
*Variants in noncoding regions*			
c.‐114_‐57del	‐	‐	‐
c.‐2C>T	‐	‐	‐
c.‐43_‐4del	‐	8	0*
c.321 + 8C>G	Intron variant	‐	‐
c.321 + 35_321 + 49del	Intron variant	2	0
c.321 + 9G>A	Intron variant	10	2
c.322‐4G>A	Intron variant	‐	‐
c.605 + 9C>T	Intron variant	‐	‐
c.605 + 54C>T	Intron variant	‐	‐
c.736‐20T>G	Intron variant	‐	‐
c.736‐6C>T	Intron variant	17	4
c.889 + 8G>C	Intron variant	57	9
*Synonymous variants*			
c.138C>T	Gly46Gly	‐	‐
c.141G>A	Thr47Thr	6	0
c.189C>T	His63His	5	3
c.309C>T	Tyr103Tyr	‐	‐
c.486C>T	Thr162Thr	‐	‐
c.603C>T	Phe201Phe	1	0
c.705C>T	Tyr235Tyr	‐	‐
c.771C>T	Thr257Thr	534	101
c.[771C>T] + [771C>T]	Thr257Thr	228	46
c.771T>C	Thr257Thr	21	6
c.[771T>C] + [771T>C]	Thr257Thr	16	9
c.804C>T	Ser268Ser	‐	‐
c.858G>C	Ser286Ser	‐	‐
c.1017C>T	Phe339Phe	0	1
*Missense variants*			
c.193G>A	Ala65Thr	‐	‐
**Reported FHH2‐ or ADH2‐causing *GNA11* variants**			
c.161C>T	Thr54Met (FHH)	2	0
c.179G>A	Arg60His (ADH)	0	1
c.179G>T	Arg60Leu (ADH)	0	4
c.196G>A	Gly66Ser (ADH)	0	1
c.446G>A	Arg149His (ADH)	0	1
c.542G>A	Arg181Gln (ADH)	0	1
c.659T>C	Phe220Ser (FHH)	1	0
c.1018G>A	Val340Met (ADH)	0	1
c.1023C>G	Phe341Leu (ADH)	0	1
**Variants of uncertain significance**			
c.259A>G	Met87Val	1	0

‐, not known, *this variant was identified in one hypocalcemic proband after functional characterization had been completed.

**Table 2:** Biochemical Features of Hypercalcemic Probands with *GNA11* VUS, Intronic Deletion, and Deletion Involving 5'UTR

					Serum				Urine
Proband	Sex of proband	Age at diagnosis	*GNA11* variant	Genetic state	Calcium (mmol/L)	Phosphate (mmol/L)	Alkaline phosphatase (U/L)	PTH	Calcium creatinine clearance ratio
19/12b	F	19 years	Met87Val	Het	2.74	0.65	104	4.6	0.005
07/14	F	54 years	c.‐43_‐4del c.321 + 35_321 + 49del	Het* Hom	2.72a	1.2c	75e	7.3i	0.01
17/09	M	19 days	c.321 + 35_321 + 49del	Hom	H	N	N	N	N

*Note*: Normal ranges^(^ ^1^ ^)^; calcium 2.10–2.60 mmol/L; phosphate 0.70–1.40 mmol/L; alkaline phosphatase activity 50–130 U/L; PTH 1.3–7.6 pmol/L; urinary calcium creatinine clearance ratio > 0.01; M, male, F, female; Exact value not available but reported to be H, high or N, not known. *The biochemical features of the additional hypercalcemic probands carrying the c.‐43_‐4del variant were not available.

### Structural and in vitro signaling studies of Met87Val Gα_11_ variant

**Fig. 2:**
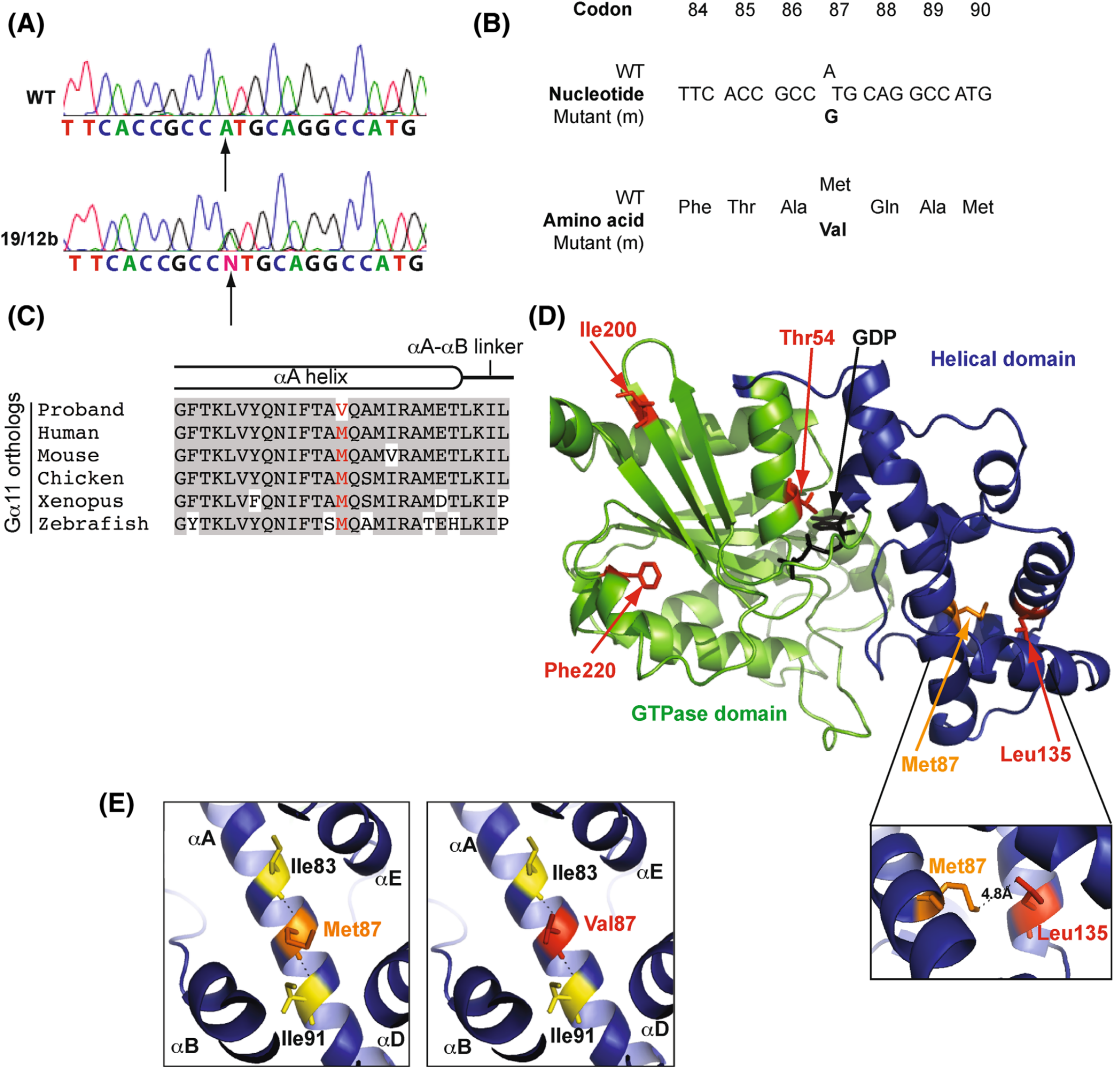
Identification of Met87Val *GNA11* variant in a hypercalcemic proband. (A) DNA sequence analyses of hypercalcemic proband 19/12b (Table 2) revealed a heterozygous A‐to‐G transition at nucleotide c.259 (arrow) within exon 2 of *GNA11*. (B) This sequence abnormality was predicted to lead to a missense amino acid substitution of Met to Val at codon 87. (C) Multiple protein sequence alignment of Gα residues within the αA helix and αA‐αB linker. The wild‐type Met87 (M) and mutant Val87 (V) residues are shown in red. Conserved residues are shaded gray. (D) Overall three‐dimensional structure of Gα_11_ protein. The reported FHH2‐causing Thr54Met, Leu135Gln, Ile200del, and Phe220Ser mutations are shown in red, and the Met87Val is shown in orange. Helical domain, blue; GTPase domain, green; guanosine diphosphate (GDP), black. Pop‐out demonstrates distance between Met87 residue and Leu135 residue. (E) Close‐up views of Gα_11_ αA helix showing wild‐type Met 87 residue (orange, left panel) and variant Val87 residue (red, right panel). Polar contacts between Met87 and Ile83 and Ile91 are shown (interrupted black lines). No change in these polar contacts are predicted with Val87 variant.

The substitution identified in exon 2 of the *GNA11* gene was due to a heterozygous c.259A>G alteration in *GNA11* that was predicted to result in a missense variant of Met87Val (Fig. 2*A*, *B*). The Met87 residue is highly conserved across vertebrate Gα11 subunit orthologs (Fig. 2*C*), and the variant, Met87Val, has been reported once out of 251,082 high‐quality genotype calls in data collated by the Genome Aggregation Database. In silico analysis using Alamut Visual Bioinformatic analyses predicted that this variant would be a VUS, SIFT and MutationTasting software^(^ ^20^, ^21^ ^)^ predicted the variant would be tolerated and benign, while MetaLR predicted the variant would be damaging.^(^ ^22^ ^)^

Homology modeling using crystal structures of the related Gα_q_ protein^(^ ^24^ ^)^ revealed that the Met87 residue was located within the αA helix of the helical domain of Gα11 (Fig. 2*D*). The Met87 residue was located away from the guanine‐nucleotide binding site but adjacent to and at a distance of 4.8 Å from the Leu135 residue, previously described to be mutated in FHH2 (Fig. 2*D*).^(^ ^6^ ^)^ Three‐dimensional homology modeling of the Gα11 protein revealed that the WT Met87 residue formed backbone polar contacts with two residues, Ile83 and Ile91, within the αA helix, which were retained by the Val87 variant (Fig. 2*E*). Furthermore, examination of the secondary structure using Spider2 predicted no differences between the helix structure in the Gα_11_ WT and Val87 variants. Thus, these structural studies suggest that the Met87Val variant is likely to be benign, and functional in vitro studies were undertaken to determine whether these in silico predictions were correct by assessing the effects of the Met87Val variant on CaSR‐mediated signaling.

**Fig. 3:**
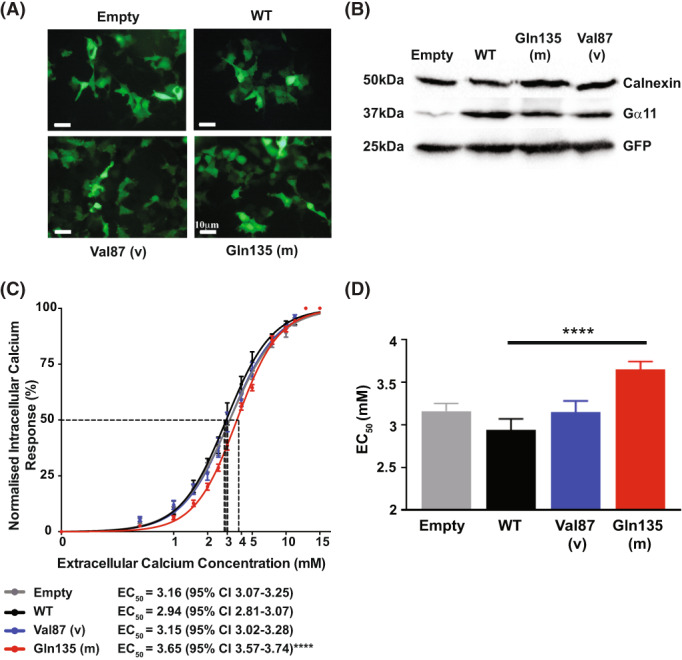
Functional characterization of, Met87 and wild‐type Val87 variant Gα11 proteins. (A) Fluorescence microscopy of HEK293 cells stably expressing CaSR (HEK293‐CaSR) and transiently transfected with wild‐type (WT), FHH2‐associated mutant (m) Gln135, or variant (v) Val87 pBI‐CMV2‐GNA11‐GFP constructs, or with vector only (empty). GFP expression in these cells indicates successful transfection and expression by these constructs. (B) Western blot analyses of whole‐cell lysates using antibodies to calnexin, Gα11, and GFP. Transient transfection with WT, Gln135, or Val87 constructs resulted in overexpression of Gα11 when compared to transfection with empty vector and normalized to calnexin expression. (C) Concentration‐response curves showing normalized Ca^2+^_i_ response to changes in [Ca^2+^]_o_ in HEK293‐CaSR cells transfected with WT, Gln135 or Val87 Gα11 constructs. The Ca^2+^_i_ responses are shown as the mean ± SEM of 4 independent transfections, and the EC_50_ values are indicated below. (D) Histograms showing Ca^2+^_o_‐induced Ca^2+^_i_ EC_50_ values with 95% confidence intervals. The FHH2‐associated Gα11 mutant Gln135 (red) led to a rightward shift of the concentration‐response curve and an increase in the EC_50_ when compared with WT Gα11 (black); *p* < 0.0001. However, the Gα11 variant Val87 (blue) did not result in a shift in the concentration response curve or significant alteration in the EC_50_ when compared with WT.

CaSR‐mediated signaling was assessed by measuring Ca^2+^_i_ responses to alterations in [Ca^2+^]_o_ of HEK293‐CaSR cells transiently transfected with pBI‐CMV2 constructs that expressed the WT Met87, variant Val87, or the previously characterized FHH2‐associated Gln135 Gα11 mutant protein. Expression of Gα11 and GFP was confirmed by fluorescence microscopy and/or Western blot analyses (Fig. 3*A*, *B*). Western blot analyses demonstrated Gα11 expression to be similar in cells transiently transfected with WT or mutant Gα11 proteins and greater than that of cells transiently transfected with the empty pBI‐CMV2 vector (Fig. 3*B*). The Ca^2+^_i_ responses in both WT and mutant Gα11‐expressing cells increased in a dose‐dependent manner following stimulation with increased [Ca^2+^]_o_ (Fig. 3*C*). Expression of the reported FHH2‐causing Gln135 Gα_11_ mutant resulted in a rightward shift of the concentration‐response curve (Fig. 3*C*), with a significantly elevated half‐maximal (EC_50_) value, compared to WT (*p* < 0.0001, Fig. 3*C*, *D*), whereas the EC_50_ value of the Val87 Gα_11_ variant protein was not significantly different from WT. Thus, the WT EC_50_ was 2.94 mM (95% confidence intervals [CIs] 2.81 to 3.07 mM), compared to 3.65 mM (95% CI: 3.57 to 3.74 mM) for Gln135‐expressing cells and 3.15 mM (95% CI: 3.02 to 3.28 mM) for Val87‐expressing cells (Fig. 3*C*, *D*). Therefore, the Val87 Gα_11_ variant does not affect Ca^2+^_i_ signaling and is best classified as a benign variant as it is unlikely to be contributing to the hypercalcemic phenotype of the patient.

### Functional assessment of a 40‐bp deletion in *GNA11* 5′UTR

**Fig. 4:**
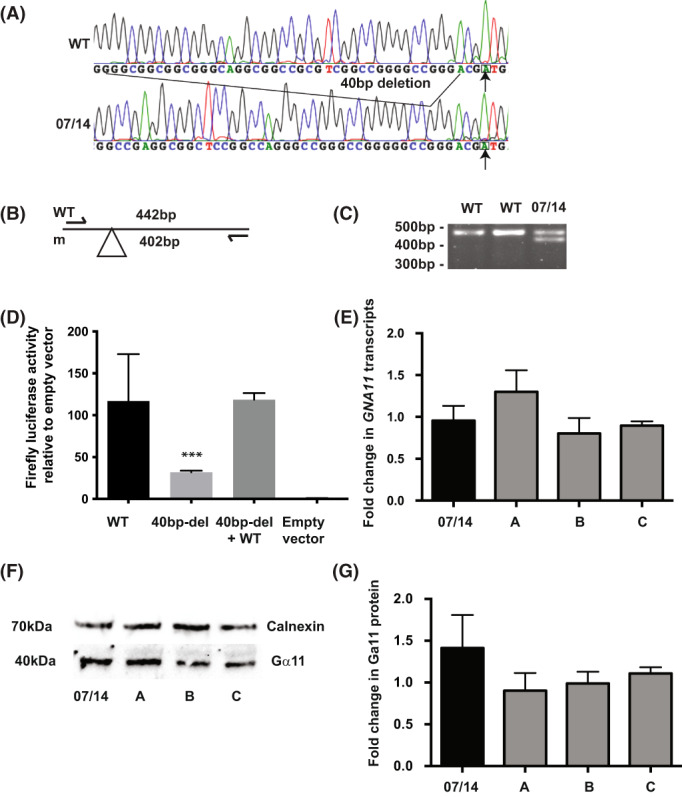
Identification of 40‐basepair (bp) deletion in 5'UTR of *GNA11* in a hypercalcemic proband. (A) DNA sequence analyses of hypercalcemic proband 07/14 (Table 2) revealed a heterozygote 40‐bp deletion within the *GNA11* 5'UTR at position −4 to −43 with respect to ATG (arrow). (B) On amplification of 5'UTR and exon 1 of *GNA11*, this sequence abnormality was predicted to result in two PCR products of 402 and 442 bp from mutant sequence (m), but only one product in the wild‐type (WT) sequence (442 bp). (C) Agarose gel electrophoresis of PCR product shown in (B) confirming presence of heterozygous deletion in 5'UTR. (D) Luciferase assay of cells transiently transfected with pGL4.10 containing WT *GNA11* promoter and either the full‐length 5'UTR (WT) or the 40‐bp deletion mutant (40 bp‐del) cloned immediately upstream of ATG translation‐initiation codon of firefly luciferase gene (*luc2*). WT luciferase activity fold‐change compared to empty pGL4.10 control = 116.8 ± 25.1, 40 bp‐del‐5'UTR luciferase activity fold‐change = 31.9 ± 0.7, cotransfection WT, and 40 bp‐del in 1:1 ratio (40 bp‐del + WT) luciferase activity fold‐change = 118.6 ± 3.2. WT versus 40 bp‐del‐5'UTR *p* = 0.0001, WT versus 40 bp‐del+WT *p* = 0.99. Statistical analyses were performed in *n* = 4 biological replicates. ***p = 0.0001. (E) Quantitative PCR (qPCR) analysis of *GNA11* transcript using total RNA from EBV‐transformed lymphoblastoid cells from proband (07/14) and three unrelated WT individuals (A, B, C). All data were normalized to geometric mean of four housekeeper genes (*CANX*, *PGK1*, *TBP1*, and *GAPDH*) and expressed as fold‐change compared to mean of WT individuals. *N* = 4 technical replicates. (F) Assessment of Gα_11_ expression by Western blot analysis using protein extracted from EBV‐transformed lymphoblastoid cell lines of proband (07/14) and 3 WT individuals (A, B, C). Calnexin was used as housekeeping protein and loading control. (G) Densitometry analysis of expression of Gα_11_ from Western blot analyses performed on four sets of independent lysates. Gα_11_ cDNA and protein expression was not significantly different in cells from the proband with 40 bp‐del‐5'UTR variant compared to WT cells.

A heterozygous 40‐bp deletion in the 5'UTR of *GNA11* encompassing positions −43 to −4 with respect to the translation‐initiation codon (ATG) was identified in eight hypercalcemic probands (Fig. 4*A*‐*C*, Table 2). The function of this deleted region is unknown but may have a role in gene expression. Therefore, despite the deletion having previously been reported (rs571847017) with a minor allele frequency (MAF) of <1%,^(^ ^29^ ^)^ we sought to assess the functional consequences of the variant on *GNA11* expression. To investigate the functional role of the 40‐bp deletion variant (40bp‐del‐5'UTR), we generated two reporter constructs, one with the WT *GNA11* promoter and WT‐5'UTR (WT‐5'UTR) (residues −1542 to −1 with respect to the translation‐initiation codon [ATG]), and one with WT *GNA11* promoter and 40bp‐del‐5'UTR (residues −1542 to −1 with respect to the translation‐initiation codon [ATG] with residues −43 to −4 deleted), cloned immediately prior to the ATG translation‐initiation codon of the Firefly luciferase gene (*luc2*) in pGL4.10. Expression of the WT‐5'UTR *GNA11* construct induced luciferase activity to 116.8 ± 25.1‐fold greater than that of the empty vector (Figure 4*D*). In contrast, cells transfected with the 40bp‐del‐5'UTR construct induced only a 31.9 ± 0.7‐fold increase in activity compared to the empty vector (Figure 4*D*). Thus, the 40‐bp deletion within the *GNA11* 5'UTR results in a >70% reduction in luciferase activity compared to transfection with the WT‐5'UTR construct (Figure 4*D*) (*p* = 0.0001, *n* = 4 experiments), thereby suggesting that it was likely pathogenic. However, cotransfection with both the WT‐5'UTR *GNA11* construct and the 40bp‐del‐5'UTR construct in a 1:1 ratio, thus mimicking the heterozygous state, resulted in no significant reduction in luciferase activity compared with transfection with the WT‐5'UTR *GNA11* construct alone (118.6 ± 3.2‐fold change relative to empty vector, *p* = 0.99, *n* = 4 experiments) (Figure 4*D*).

To further assess the effect of this variant in vivo in the heterozygous state, we undertook qPCR analyses on RNA extracted from EBV‐transformed lymphoblastoid cells from a proband with the 40bp‐del‐5'UTR variant (07/14, Table 2) and three unrelated unaffected individuals that did not have the 5'UTR variant. This demonstrated that the 40bp‐del‐5'UTR variant had no effect on *GNA11* mRNA levels (Fig. 4*E*) and therefore had no effect on *GNA11* transcription. To determine whether the 40bp‐del‐5'UTR variant reduced translational efficiency, protein was extracted from EBV‐transformed lymphoblastoid cell lines from a proband with the 40bp‐del‐5'UTR variant and the three WT control unaffected individuals. Western blot analyses revealed no difference in Gα_11_ protein expression in the affected patient cells compared to cells from three control individuals (Fig. 4*F*, *G*). Furthermore, following functional characterization of this variant, the c.‐43_‐4del variant was identified in one hypocalcemic proband (Table 1), thereby indicating that this 40bp‐del‐5'UTR was unlikely to be contributing to alterations in calcium homeostasis. Therefore, the *GNA11* 40bp‐del‐5'UTR variant is best classified as a benign variant, as it has no effect on transcription or translation of Gα_11_ and occurs in hypercalcemic and hypocalcemic individuals.

### Characterization of 15‐bp intron 3 deletion (c.321 + 35_321 + 49del) in *GNA11*

**Fig. 5:**
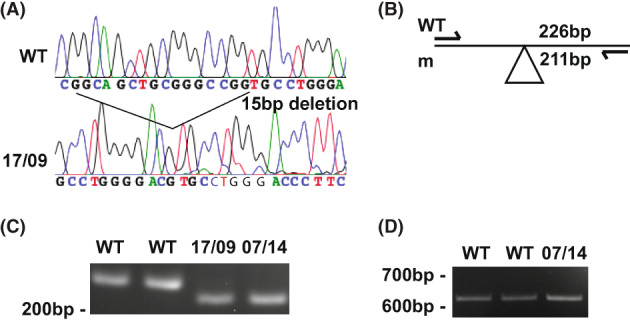
Identification of 15‐bp deletion in *GNA11* intron 3 (c.321 + 35_321 + 49del) in two unrelated hypercalcemic probands. (A) DNA sequence analyses revealed homozygous 15‐bp deletion at position +35 in *GNA11* intron 3 of two unrelated hypercalcemic probands (17/09 and 07/14). (B) On amplification of the exon 3–intron 3 boundary including 59 exonic bp and 167 intronic bp, this sequence abnormality was predicted to result in two products of 226 and 211 bp from mutant sequence (m), but only one product in wild‐type (WT) sequence (226 bp). (C) Agarose gel electrophoresis of PCR product shown in (B) confirming presence of homozygous deletion in intron 3 in both probands (07/14 and 17/09). (D) Agarose gel electrophoresis demonstrated no alteration in PCR product length for WT cDNA and proband 07/14 cDNA using primers spanning between *GNA11* exon 1‐2 (c.119‐140) and exon 5‐6 (c.733‐753).

A homozygous 15‐bp deletion in intron 3 of *GNA11* was identified in two unrelated hypercalcemic probands (07/14 and 17/09, Tables 1 and 2). This variant has a minor allele frequency of <0.1% (c.321 + 35_321 + 49del, deletion of residues 35–49 with respect to the start on intron 2–3, rs145870964) (Fig. 5*A*‐*C*).^(^ ^29^ ^)^ To investigate whether this variant resulted in a splicing defect, RNA was extracted from EBV‐transformed lymphoblastoid cells established from one proband (07/14) and RT‐PCR analysis performed using primers at the exon 1–2 (c.119‐140) and exon 5–6 (c.733‐753) boundaries. However, RT‐PCR analysis demonstrated no alteration in the PCR product length between cDNA from the proband and two unrelated WT control individuals (Fig. 5*D*). Furthermore, sequence analysis of the cDNA product revealed no alteration in DNA sequence when comparing the proband with cDNA from WT individuals (data not shown). Therefore, the c.321 + 35_321 + 49del deletion had no effect on splicing and is best classified as a benign variant.

## Discussion

Over a 10‐year period, we identified 37 different *GNA11* variants in >1200 probands referred for investigation of a genetic cause of their hypercalcemia or hypocalcemia. However, only nine of these variants (Thr54Met, Arg60His, Arg60Leu, Gly66Ser, Arg149His, Arg181Gln, Phe220Ser, Val340Met, Phe341Leu), occurring in 13 individuals, were found to be likely disease causing. Our studies highlight that not all rare variants identified in the *GNA11* gene in patients are the cause of the hyper‐ or hypocalcemic phenotype. Thus, the missense variant, Met87Val, identified in an individual with hypercalcemia had only been reported once in >250,000 alleles, and SIFT and Mutations Tasting protein prediction software indicated that this variant would be benign, while the MetaLR tool predicted this variant would be damaging. The Met87 residue lies only 4.8 Å from Leu135, the mutation of which to Gln is known to cause FHH2.^(^ ^6^ ^)^ However, functional analyses demonstrated that the Met87Val Gα_11_ variant had no effect in vitro on CaSR‐mediated signaling (Fig. 3). This is consistent with modeling studies that indicated that the Met87Val variant had little effect on the Gα_11_ protein structure. Met87Val is located within an α‐helix of the Gα_11_ helical domain and is not predicted to disrupt the structure of Gα_11_ (Fig. 2). In contrast, the four mutations previously identified in Gα_11_ that are associated with FHH2 have been shown to affect regions critical for G‐protein activation, effector coupling, or GTP‐GDP binding.^(^ ^4^, ^5^, ^6^ ^)^ Thus, it is likely that Gα_11_ variants that are identified in structural domains known to have critical roles in G‐protein activation are disease‐causing, and structural modeling may be a better predictor of likely pathogenicity than protein prediction programs. This further highlights the limitations of bioinformatics and protein prediction software, which may be inaccurate,^(^ ^22^ ^)^ as we previously reported for AP2σ variants.^(^ ^30^ ^)^

In addition to this missense variant, two rare variants in the noncoding regions of *GNA11*, a heterozygous 40‐bp deletion in the 5'UTR and a homozygous 15‐bp intron deletion, were identified in two probands that had a phenotype of FHH and were functionally characterized. In vitro characterization of the effect of the 5'UTR deletion by luciferase assay demonstrated that the 40‐bp deletion reduced luciferase expression compared with the WT‐5'UTR (Fig. 4). However, cotransfection with both the WT‐5'UTR *GNA11* construct and the 40bp‐del‐5'UTR construct at a 1:1 ratio, thereby mimicking the heterozygous state, resulted in no significant reduction in luciferase activity compared with transfection with the WT‐5'UTR *GNA11* construct alone. Furthermore, studies of Gα11 expression in EBV‐transformed lymphoblastoid cells from the affected individual demonstrated no significant differences compared with cells from unaffected individuals (Fig. 4). Moreover, this variant was subsequently identified in one hypocalcemic proband. Thus, our results suggest that the *GNA11* 40bp‐del‐5'UTR variant does not alter protein expression in vivo in the heterozygous state, consistent with previous studies of the *GNA11* promoter and 5'UTR region that demonstrated that the essential regulatory regions for *GNA11* promoter activity were located between nucleotides −805 and −177.^(^ ^31^ ^)^ However, the transcription start sites of *GNA11* are poorly understood,^(^ ^31^ ^)^ and the 5'UTR may contain regulatory elements for translation that have not yet been described. Our results suggest that this variant in the homozygous state may result in a reduction in Gα11 expression. Studies of the homozygous 15‐bp intronic deletion in *GNA11* identified in a patient with a FHH phenotype also indicate that this is a benign variant. Thus, RT‐PCR analysis of exons 2–5 of the *GNA11* gene in EBV‐transformed lymphoblastoid cells from the patient showed the cDNA products were not different when compared with those from WT (control) individuals (Fig. 5). These findings indicate that the 15‐bp deletion is likely a nonfunctional polymorphism.

Our results, showing a low frequency of pathogenic *GNA11* variants, are consistent with the low frequency of *GNA11* variants observed in the Genome Aggregation Database. Indeed, the observed frequency of missense and predicted loss‐of‐function *GNA11* variants is significantly lower than that expected in the ExAC database^(^ ^32^ ^)^ (expected versus observed *GNA11* variants: missense = 252.5 versus 84, *Z*‐score 3.77; predicted loss of function = 15.9 versus 2, loss of function observed/expected upper bound fraction 0.4). This suggests that Gα_11_ variants are not well tolerated and further indicates the critical role of the affected residues in calcium homeostasis and signaling of multiple GPCRs.^(^ ^33^ ^)^

In summary, we identified 37 different *GNA11* variants in 1226 probands undergoing investigation for a genetic cause of their hypercalcemia or hypocalcemia over a 10‐year period. However, only nine of these variants were found to be disease‐causing, and our studies highlight the importance of comprehensive in silico and in vitro analysis of *GNA11* genetic variants to prevent misdiagnoses.

## Author Contributions

**Sarah A. Howles:** Methodology; conceptualization; investigation; formal analysis; writing – original draft; writing – review and editing; validation. **Caroline M. Gorvin:** Conceptualization; methodology; supervision; formal analysis; validation; investigation; writing – original draft; writing – review and editing. **Treena Cranston:** Investigation; formal analysis; data curation; writing – review and editing. **Angela Rogers:** Investigation; validation; formal analysis; writing – review and editing. **Anna K. Gluck:** Investigation; validation; formal analysis; writing – review and editing. **Hannah Boon:** Investigation; validation; formal analysis; data curation; writing – review and editing. **Kate Gibson:** Investigation; validation; formal analysis; data curation; writing – review and editing. **Mushtaqur Rahman:** Investigation; formal analysis; writing – review and editing. **Allen Root:** Investigation; formal analysis; writing – review and editing. **M. Andrew Nesbit:** Conceptualization; methodology; investigation; validation; formal analysis; supervision; writing – review and editing. **Fadil M. Hannan:** Conceptualization; methodology; formal analysis; supervision; writing – original draft. **Rajesh V. Thakker:** Conceptualization; methodology; formal analysis; supervision; funding acquisition; writing – original draft; writing – review and editing.

## Disclosures

RVT has received grants from Novartis Pharma AG, Novo Nordisk, and the Marshall Smith Syndrome Foundation for unrelated studies. No other authors have conflicts of interest.

### Peer Review

The peer review history for this article is available at https://www.webofscience.com/api/gateway/wos/peer-review/10.1002/jbmr.4803.

## Data Availability

The data that support the findings of this study are available from the corresponding author upon reasonable request.
